# Immunotherapies and Combination Strategies for Immuno-Oncology

**DOI:** 10.3390/ijms21145009

**Published:** 2020-07-15

**Authors:** Cody Barbari, Tyler Fontaine, Priyanka Parajuli, Narottam Lamichhane, Silvia Jakubski, Purushottam Lamichhane, Rahul R. Deshmukh

**Affiliations:** 1 OMS Students, School of Osteopathic Medicine, Lake Erie College of Osteopathic Medicine (LECOM), 5000 Lakewood Ranch Blvd, Bradenton, FL 34211, USA; CBarbari81565@med.lecom.edu (C.B.); TFontaine56585@med.lecom.edu (T.F.); 2 Department of Internal Medicine, Southern Illinois University, Springfield, IL 62702, USA; priyankaparajulimd@gmail.com; 3 Department of Radiation Oncology, University of Maryland, School of Medicine, Baltimore, MD 21201, USA; NaruLamichhane@umm.edu; 4 Department of Biostatistics, University of Florida, Gainesville, FL 32611, USA; si.jakubski@gmail.com; 5 School of Dental Medicine, Lake Erie College of Osteopathic Medicine (LECOM), 4800 Lakewood Ranch Blvd, Bradenton, FL 34211, USA; 6 School of Pharmacy, Lake Erie College of Osteopathic Medicine (LECOM), 5000 Lakewood Ranch Blvd, Bradenton, FL 34211, USA

**Keywords:** checkpoint inhibition, immunotherapy, chemoresistance, combination therapy, radiation therapy, adoptive cell transfer, chemotherapy

## Abstract

The advent of novel immunotherapies in the treatment of cancers has dramatically changed the landscape of the oncology field. Recent developments in checkpoint inhibition therapies, tumor-infiltrating lymphocyte therapies, chimeric antigen receptor T cell therapies, and cancer vaccines have shown immense promise for significant advancements in cancer treatments. Immunotherapies act on distinct steps of immune response to augment the body’s natural ability to recognize, target, and destroy cancerous cells. Combination treatments with immunotherapies and other modalities intend to activate immune response, decrease immunosuppression, and target signaling and resistance pathways to offer a more durable, long-lasting treatment compared to traditional therapies and immunotherapies as monotherapies for cancers. This review aims to briefly describe the rationale, mechanisms of action, and clinical efficacy of common immunotherapies and highlight promising combination strategies currently approved or under clinical development. Additionally, we will discuss the benefits and limitations of these immunotherapy approaches as monotherapies as well as in combination with other treatments.

## 1. Introduction

Cancer is the second leading cause of death in the United States and continues to significantly affect populations worldwide [1]. Traditional therapies for cancer have been based on the patient’s tumor type and stage with treatment variations of surgery, radiation, and/or chemotherapy [2]. However, even with the advancements in medicine, many patients progress and succumb to cancer. A relatively new field of cancer therapy includes immunotherapy. Immunotherapy aims to harness the immune system’s ability to recognize, target, and destroy cancerous cells [3]. Cancer immunotherapy now encompasses many therapeutic agents, each with their own unique targets and mechanisms of actions. These therapies aim to utilize various components of the immune system, acting on distinct steps of the immune response to modulate natural defenses against tumor cells [3]. While many immunotherapy agents can be effective as a monotherapy option, the primary interest as of late has been to find clinical evidence of effective combinations with traditional therapies or other immunotherapy agents such that maximum therapeutic benefits can be obtained in a wide range of patients [4]. Each specific combination may have a unique mechanistic synergism, which offers a complicated yet potentially rewarding feat in the field of immuno-oncology.

Cancer evades the detection and assault by the immune system through a range of escape mechanisms, allowing unopposed growth and proliferation over time [4]. Immunosuppressive cytokines and inhibitory proteins, such as transforming growth factor-β (TGF-β), interleukin (IL)-4, programmed cell death 1 (PD-1), and programmed death ligand 1 (PD-L1), allow tumor cells to escape immune attack [5]. Various immunotherapy modalities (discussed below) have different ways of modulating natural defenses against tumor cells. Typically, this involves checkpoint blockades, molecular modification of cell regulatory pathways, or direct modification of host T cells, all of which aim to generate sustainable T cell responses against cancerous cells [6].

The efficacy of immunotherapy relies on its ability to produce sustainable T cell responses. Regulatory pathways and checkpoint molecules control activation, progression, and sustainability of immune responses, thus dictating the progression or control of cancer growth. Checkpoint inhibitors have had great success in treatments of cancers such as metastatic melanoma, non-small cell lung cancer (NSCLC), and renal cancers [7]. Over the last decade, while there have been hundreds of immunotherapy trials, many of which are currently in progress (clinicaltrials.gov), only some have led to approval of some of these inhibitors for treatment of different cancers [8,9,10]. Inhibitors of checkpoint molecules such as PD-1, PD-L1, cytotoxic T- lymphocyte-associated protein 4 (CTLA-4), T cell immunoglobulin and mucin domain 3 (TIM3), lymphocyte-activation gene 3 (LAG3), and V-domain Ig suppressor of T cell activation (VISTA) have and will continue to dramatically change the landscape of immunotherapy. Checkpoint inhibitors essentially function to “release the brakes” on the immune system by blocking ligands or receptors that prevent activation and functions of immune effectors. While checkpoint inhibitors as monotherapies have generated impressive results in subsets of different cancer patients, they have remained ineffective in many more. To overcome this challenge, combination therapy with multiple immune checkpoint inhibitors is frequently studied [11]. However, research is also underway to determine the efficacy of combining checkpoint blockade with targeting of costimulatory receptors. The introduction of two agents that function to “release the brakes” (checkpoint inhibitors) and “step on the gas” (costimulatory receptors) could work collectively to mount a stronger immune response against cancers. Combinations such as these are currently under clinical investigation and discussed throughout this manuscript.

Combination of immunotherapies with traditional therapies, such as chemotherapies, have also shown clinical benefits in certain cancers that previously had a poor prognosis. An impressive example of immunotherapy efficacy is treatment for NSCLC. NSCLC was historically treated with cytotoxic chemotherapy agents (such as cisplatin or carboplatin) or a checkpoint blockade as monotherapies [12,13,14]. However, on 30 October 2018, the U.S. Food and Drug Administration (FDA) approved the anti-PD-1 antibody pembrolizumab in combination with chemotherapy as a first line treatment for NSCLC. In a phase III trial of 559 patients, those who received the combination therapy (pembrolizumab plus carboplatin and either paclitaxel or nab-paclitaxel) had a median overall survival of 15.9 months compared to 11.3 months in those treated with chemotherapy alone (*p*-value < 0.001, confidence interval (CI) 95%, hazard ratio (HR) 0.64). These benefits were seen regardless of the amount of PD-L1 tumor expression in these patients [15]. There are a variety of other promising immune-oncology combination strategies that are under exploration [16,17]. The combination of immunotherapy with chemotherapy has become a standard option in some cancers [18]. Chemotherapy drugs such as cyclophosphamide (an alkylating agent that affects DNA, RNA, and protein synthesis) or fludarabine (a DNA synthesis inhibitor) can be used prior to administration of immunotherapy as a preconditioning regimen [19]. Recently, these drugs have been FDA approved for use in patients with chronic lymphocytic leukemia (CLL) in conjunction with the monoclonal antibody (mAb) rituximab (anti-CD20 mAb) [20]. These drugs act to deplete regulatory T cells prior to adoptive T cell therapy (such as chimeric antigen receptor (CAR) T cell therapy), which extends the lifespan and utility of the modified infused T cells [19]. While each combination may have a unique mechanistic synergism, much work remains to be done to elucidate the dose, sequence, and timing of the combinations that result in optimal efficacy with manageable toxicities.

Unpredictable response rates for PD-1/PD-L1 inhibitors led investigations to explore potential biomarkers present in the tumor microenvironment. In general, it is accepted that the extent of PD-L1 expression correlates with better therapeutic response rates for anti-PD-1 or anti-PD-L1 therapies [21]. Pembrolizumab (anti-PD-1 mAb) for NSCLC is indicated as a monotherapy if the patient has greater than 50% expression of PD-L1 on tumor cells [15]. However, it should be noted that there are challenges in determining the predictive value of PD-L1 expression when combination therapy is used. Limitations include therapy-induced alteration of the immune microenvironment and heterogeneity of PD-L1 expression [22]. Pembrolizumab was the first FDA-approved drug on the basis of two common biomarkers known as mismatch repair deficient (dMMR) or microsatellite instability-high (MSI-H) for patients with metastatic solid tumors [23]. This approach represents the potential for a future generation of drugs classified towards biomarkers of cancer regardless of the primary location of the tumor [24].

The cost of immunotherapy treatments is an additional challenge to be considered. Immune checkpoint inhibitor therapies currently have an average cost of nearly $150,000 per year [24]. Combination therapies, such as ipilimumab (CTLA-4 inhibitor) and nivolumab (PD-1 inhibitor), which are commonly used for advanced melanomas, intuitively bear more cost. Patients can expect to pay upwards of a quarter million dollars for each year of treatment [24]. Newer modalities such as adoptive cellular therapy (ACT) treatments may carry even higher price tags. While health insurance may take a major financial strain off patients and families, out-of-pocket costs continue to climb. Although immunotherapies are considerably expensive, it is important to note that the durability of response may outweigh the cost of lengthier, less effective chemotherapy treatments. A solution currently being investigated is targeting biomarkers to isolate specific patient populations that have a chance to experience greater benefits from these treatments and lead to a reduced overall economic burden [25,26]. There are several challenges that lie ahead for personalized combinatory immunotherapies. Oncologic therapy’s standard of care is changing rapidly, and immunotherapy is becoming one of the frontline treatments in modern medicine. Below, we describe different types of immunotherapies, discuss their advantages and limitations, and evaluate the available research and outlook for combination strategies. 

## 2. Types of Immunotherapy: Their Combinations and Limitations

### 2.1. Checkpoint Inhibitors

#### 2.1.1. Programmed Cell Death-1 (PD-1) Blockade and Combinations 

The checkpoint receptor PD-1 is expressed on various immune cells, such as T cells, B cells, macrophages, natural killer (NK) cells, and dendritic cells, as well as cancer cells. Major ligands for PD-1 include PD-L1 (B7-H1) and PD-L2 (B7-DC). These ligands are expressed on various immune cells, including antigen presenting cells and are often overexpressed on tumor cells [27]. Upon binding to its ligands, PD-1 is responsible for the inability of antigen presenting cells (APCs) to process and present tumor antigens and for the anergic state of T cells. Binding of PD-1, on T cells, to its ligand PD-L1 results in recruitment of the phosphatase SHP-2 (Src homology 2 domain-containing protein tyrosine phosphatase), leading to dephosphorylation of proximal signaling kinases of T cells [28]. In T cells, this signaling cascade results in inhibition of T cell responses, cytokine production, cytotoxic activity, and proliferation, as well as promotion of T cell apoptosis. Blockade of this signaling cascade with antagonist antibody against PD-1 or PD-L1 can reinvigorate the T cells. In an early study, a fully human IgG4 monoclonal antibody (mAb) against PD-1, known as nivolumab, showed impressive response rates in various tumors [27]. Since then different PD-1 and PD-L1 inhibitors have been successfully tested and FDA approved against various tumors [8,9,10]. 

Despite impressive response rates, many patients do not benefit from PD-1/PD-L1 blockade as monotherapies, owing to different innate and adaptive resistance mechanisms and oncogenic pathways [11,29]. This has prompted testing of a combination of PD-1/PD-L1 blockade with other therapeutic modalities including additional checkpoint blockade, chemotherapies, targeted therapies, and radiation therapies to improve efficacies of checkpoint blockade. As a means of improving efficacies, many studies have evaluated the safety and efficacy of combination of PD-1/PD-L1 blockade with targeted- or chemotherapies. In a study of relapsed or refractory Hodgkin’s lymphoma patients who did not benefit from prior PD-1 blockade monotherapy, a combination of PD-1 inhibitors (nivolumab or pembrolizumab) with chemotherapy (in different combinations of vinblastine, gemcitabine, bendamustine, liposomal doxorubicin, ifosfamide, carboplatin, etoposide, cisplatin, cytarabine, dexamethasone, vinorelbine) resulted in an overall response rate (ORR) of 86%; while chemotherapy alone resulted in an ORR of 59% [30]. It is important to note that, in this study, the combination group had relatively younger patients and less patients with progressive disease after prior anti-PD-1 therapy. Similarly, a study in advanced pancreatic cancer patients evaluated the differences in efficacy of chemotherapy (*n* = 36) vs. chemotherapy in combination with PD-1/PD-L1 blockade (*n* = 22) [31]. The combination group had significantly higher overall survival compared to chemotherapy alone group (median survival: 18.1 vs. 6.1 months; *p* = 0.021). While no significant difference in the ORR was observed; the progression-free survival was 3.2 months compared to 2.0 months for chemotherapy alone group (*p* = 0.041) [31]. Similarly, in advanced biliary tract cancer patients, chemotherapy (gemcitabine-based, paclitaxel-albumin-based, oxaliplatin + tegafur, or other regiments) plus PD-1 blockade (pembrolizumab or nivolumab) resulted in an overall survival (OS) of 14.9 months compared to 4.1 and 6.0 months, respectively for PD-1 blockade alone and chemotherapy alone [32]. In this study, the progression-free survival (PFS) for combination therapy was 5.1 months compared to 2.2 months for PD-1 blockade alone (*p* = 0.014). In a large phase III trial in patients with triple-negative breast cancer, a combination of atezolizumab (a fully humanized IgG1 against PD-L1) with nab-paclitaxel was shown to result in PFS of 7.2 months compared to 5.5 months for placebo plus nab-paclitaxel (*p* = 0.002) [33]. The median OS was 21.3 months for combination compared to 17.6 months for placebo plus nab-paclitaxel alone. The OS was even higher (25 months vs. 15.5 months) when patients were stratified by PD-L1 positivity for tumors. Based on the efficacy results from a double-blind, placebo-controlled, phase III trial, atezolizumab plus carboplatin and etoposide have been FDA approved for first-line treatment of adult patients with extensive-stage small cell lung cancer [34]. A combination of poly(ADP-ribose) polymerase (PARP) inhibitors with PD-L1 inhibitor (olaparib + durvalumab) has also been tested, with results showing improved efficacies of combination treatments in germline BRCA-mutated platinum-sensitive relapsed ovarian cancer patients [35] and patients with relapsed gastric cancer [36] in the MEDIOLA study. Interestingly, some chemotherapies have been shown to increase the expression of PD-1/PD-L1, hence contributing to immunosuppression and poor responses to chemotherapies alone [37,38,39]. This may explain, in part, the improved responses observed with a combination of chemotherapies and PD-1/PD-L1 blockade.

There are several PD-L1 inhibitor combination studies that are currently recruiting for phase I and II trials. A randomized phase II (NCT03959293) study with a “stop and go” analysis is evaluating durvalumab with FOLFIRI (folinic acid (leucovorin) + fluorouracil + irinotecan) vs. tremelimumab (a fully human mAb against CTLA-4) and durvalumab with FOLFIRI for advanced gastric adenocarcinoma [40]. Another study (NCT02349633) is aiming to look at different cohort combinations of anti-PD-1/PD-L1 in previously treated NSCLC patients with epidermal growth factor receptor (EGFR) mutation [41]. Cohorts of the study will compare combination of their study drug: PF-06747775 (EGFR inhibitor) in combination with palbociclib (a cyclin-dependent kinase (CDK) 4 and 6 inhibitor) (cohort 2) and avelumab (PD-L1 inhibitor) (cohort 3). Results for phase II were estimated to be released sometime after 31 March 2020, but no results have been published on trials website at the time this review was written. Similar to these, many other studies are ongoing to evaluate combinations of PD-1/PD-L1 blockade with targeted and chemotherapies. Results from these studies are eagerly awaited.

#### 2.1.2. Cytotoxic T-Lymphocyte-Associated Protein-4 (CTLA-4) Blockade and Combinations

**Figure 1:**
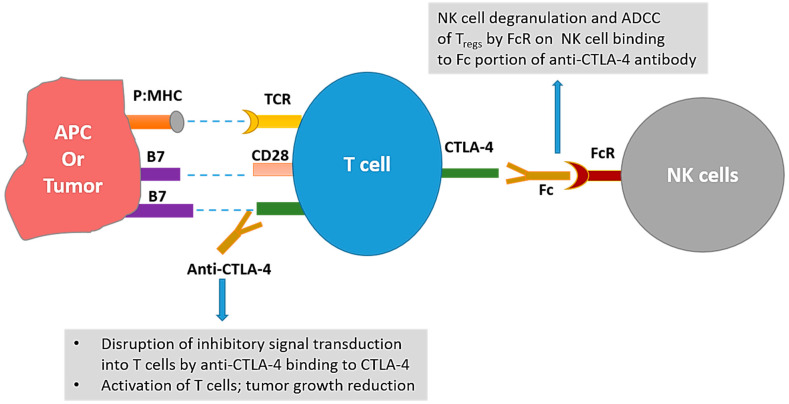
Mechanisms of anti-tumor actions of cytotoxic T- lymphocyte-associated protein 4 (CTLA-4) targeting antibodies. Interaction of CTLA-4 on T cell with B7-1 or B7-2 on antigen presenting cells (APCs) leads to inhibition of T cell activity. Disruption of this interaction with anti-CTLA-4 antibody leads to activation of T cells and subsequent tumor growth inhibition. Additionally, interaction between the Fc portion of the anti-CTLA-4 antibodies and the Fc receptors (FcR) on natural killer (NK) cells may lead to antibody dependent cellular cytotoxicity (ADCC) of T_regs_ leading to depletion of T_regs_ in mice. p:MHC = peptide:major histocompatibility complex.

Similar to PD-1, CTLA-4 is a checkpoint of the immune system responsible for the negative regulation of T cells. CTLA-4 is a CD28 homolog that has much higher affinity for B7 molecules than CD28. This CTLA-4:B7 interaction not only leads to inhibitory signaling in T cells, but also prevents the costimulatory signal transduction by outcompeting the CD28:B7 interactions [42]. While the culminating negative effects of both PD-1 and CTLA-4 on T cell activity are similar; there are some differences between the two: (i) PD-1 limits the T cell responses later in the immune response compared to CTLA-4, which limits the T cell responses early in the immune response; (ii) different combinations of molecules are involved in signal transduction of CTLA-4 vs. PD-1; and (iii) in addition to some shared effects, blockade of these molecules can have distinct effects on different cells [42,43,44]. The blockade of CTLA-4 has been thought to work by not only activating the T cells, but also by depletion of regulatory T cells (T_regs_). Anti-mouse CTLA-4 antibodies have been shown to effectively reduce T_regs_ in the tumor microenvironment [45,46]; although a recent report suggests that T_reg_ depletion does not occur with anti-CTLA-4 therapy (ipilimumab or tremelimumab) in humans and that opportunity exists to modify F_c_ portions of the CTLA-4 antibodies to achieve T_reg_ depletion [47]. Figure 1 reflects the proposed mechanisms of action of CTLA-4 blockade.

In one of the largest phase III trials of CTLA-4 checkpoint blockade to date, ipilimumab was tested in patients with previously treated malignant melanoma [48]. The randomized double-blind study had a pool of 676 patients with stage III–IV melanoma (unresectable). In total, 403 of these patients were randomly assigned to receive 3 mg/kg ipilimumab combined with a monomeric antigen gp100 HLA-A0201 vaccine. The remaining patients received 3 mg/kg ipilimumab alone (*n* = 137) or the gp100 HLA-A0201 vaccine alone (*n* = 136). The median overall survival for ipilimumab monotherapy was 10.1 months compared to 10.0 months and 6.4 months, respectively for patients receiving ipilimumab with the gp100 vaccine and gp100 vaccine alone [48]. In this study, grade 3 or 4 immune-related adverse events were experienced by 10–15% of patients receiving ipilimumab. Based on this pivotal trial, the FDA approved ipilimumab in 2011 for treatment of patients with inoperable or metastatic melanoma [49].

Similar to PD-1 blockade, various combinations with anti-CTLA-4 have been evaluated. In a phase III trial of treatment-naïve patients with advanced melanoma, five-year survival for patients receiving ipilimumab plus dacarbazine was 18.2% compared to 8.8% for patients receiving placebo plus dacarbazine [50]. In this study, patients without dose limiting toxicities from week 12 through week 24 received ipilimumab or placebo (every 12 weeks) as maintenance therapy [50]. In a randomized phase II trial evaluating the efficacy of ipilimumab plus sargramostim (GM-CSF) vs. ipilimumab alone in metastatic melanoma patients, it was observed that median overall survival (OS) for ipilimumab plus sargramostim was 17.5 months compared to 12.7 months for ipilimumab alone; although median progression-free survival for both groups was 3.1 months [51]. At the median follow-up of 13.3 months, the one-year survival rate for combination was 68.9% as compared to 52.9% for ipilimumab monotherapy [51]. Survey of clinicaltrials.gov shows that while some trials evaluating the combinations with anti-CTLA-4 have failed or halted, there are many ongoing trials aiming to evaluate safety and efficacy against various tumor types. Most of these trials also include PD-1/PD-L1 blockade in the combinations.

### 2.2. Combination of Anti-PD-1/PD-L1 and Anti-CTLA-4 

As mentioned above, monotherapy with nivolumab or ipilimumab has shown to be promising in recent years due to increasing durability of treatment and high response rates in several types of cancer in select patients. However, despite these efficacies, there are still patients who do not benefit from these treatments. This led researchers to investigate combinatory therapies that aim to prevent resistance mechanisms and target non-redundant inhibitory signaling to provide sustainable tumor regression. One combination often studied is the combination of PD-1/PD-L1 blockade with CTLA-4 blockade. In the study, CheckMate-142, by Overman et al. on metastatic colorectal cancer (previously treated patients whose tumors are DNA mismatch repair deficient (dMMR)/microsatellite instability-high (MSI-H)), nivolumab was shown to have higher response rates in combination with ipilimumab [52]. While nivolumab as a monotherapy had an overall survival of 73% at 12 months, it was 85% for nivolumab in combination with ipilimumab [52]. In this study, any grade treatment-related adverse events (TRAEs) for this combination therapy was 73% compared to 70% for nivolumab monotherapy. Other studies, however, have shown significantly higher TRAEs with the combination [53,54]. In a randomized phase II study, combination of tremelimumab (anti-CTLA-4) and durvalumab (anti-PD-L1) was tested in patients with metastatic refractory colorectal cancer [55]. The median OS was 6.6 months for combination compared to 4.1 months for the best supportive care (BSC) available; although no significant differences in PFS was observed (1.8 months vs. 1.9 months). Additionally, significantly higher proportions of patients in the combination group experienced grade 3/4 adverse events compared to the BSC (64% vs. 20%) [55].

In a phase II randomized study of high-risk resectable melanoma patients (*n* = 23), neoadjuvant nivolumab was compared with neoadjuvant combination of ipilimumab and nivolumab [56]. The combined therapy resulted in an overall response rate of 73% compared to 25% for nivolumab monotherapy. The pathologic complete response rate was 45% for combination compared to 25% for nivolumab monotherapy; however the grade 3 toxicities were significantly higher in combination group vs. nivolumab monotherapy (73% vs. 8%) [56]. In this study, higher lymphoid infiltrates were associated with response to therapies [56]. Furthermore, 11 out of 11 patients in combination group were still alive at the median follow-up of 15.6 months [56]. Anti-CTLA-4 plus anti-PD-1 in a neoadjuvant vs. adjuvant setting has also been studied, with results showing higher clinical activity with the neoadjuvant treatment; although toxicities were also higher [57]. Larger studies need to be conducted to effectively compare the differences in the survival benefits of this combination in an adjuvant vs. neoadjuvant setting.

Different combinations of doses of anti-CTLA-4 and anti-PD-1 have also been studied. In a multicenter, open-label, phase I/II study (CheckMate 032), combination of ipilimumab and nivolumab was evaluated at different doses in platinum-pretreated patients with unresectable locally advanced or metastatic urothelial carcinoma [58]. Three cohorts were included in the analysis: 3 mg/kg nivolumab with 1 mg/kg ipilimumab (NIVO3 + IPI1), nivolumab only (NIVO3), and 3 mg/kg of ipilimumab with 1 mg/kg nivolumab (NIVO1 + IPI3) [58]. With an objective response rate of 38%, NIVO1 + IP3 was found to be superior to NIVO3 and NIVO3 + IPI1, which had respective objective response rates of 25.6% and 26.9%. For NIVO3, NIVO3 + IPI1, and NIVO1 + IPI3, the median PFS was 2.8 months, 2.6 months, and 4.9 months, respectively. In the NIVO1 + IPI3 group, 39.1% of patients experienced grade 3/4 adverse events compared to 26.9% and 30.8%, respectively for NIVO3 and NIVO3 + IPI1 [58]. Phase III study (CheckMate 901) will evaluate the efficacy of NIVO1 + IPI3 in patients with untreated inoperable or metastatic urothelial cancer, with the study completion date estimated to be August 2024 (NCT03036098). Similarly in a randomized, double-blind, phase III study (CheckMate 511), NIVO1 + IPI3 was compared with NIVO3 + IPI1 in patients with previously untreated, unresectable stage III or IV melanoma [59]. In this study, no significant differences in objective response rates were observed between the groups (45.6% for NIVO3 + IPI1 vs. 50.6% in the NIVO1 + IPI3) at a minimum follow-up of 12 months; however, similar to other studies, the grade 3 to 5 adverse events were significantly higher for NIVO1 + IPI3 group (48% vs. 34%) [59]. Both NIVO1 + IPI3 and NIVO3 + IPI1 have been FDA approved for treatment of different cancers. These results demonstrate that optimal dose combinations of nivolumab and ipilimumab may be dependent on disease type, disease stage, and prior treatments, among others.

Based on results from different pivotal trials, combinations of nivolumab and ipilimumab have been FDA approved, among others, for patients with hepatocellular carcinoma (HCC) who have been previously treated with sorafenib [60], for patients with intermediate or poor risk, previously untreated advanced renal cell carcinoma [61], for patients with metastatic non-small cell lung cancer whose tumors express PD-L1 (≥1%) and have no EGFR or anaplastic lymphoma kinase (ALK) genomic tumor aberrations [62], and patients with microsatellite instability-high (MSI-H) or mismatch repair–deficient (dMMR) metastatic colorectal cancer that progresses during or after treatment with a fluoropyrimidine, oxaliplatin, and irinotecan-based chemotherapies [63].

Furthermore, multiple studies are evaluating the safety and efficacy of combination of ipilimumab + nivolumab with various chemotherapies in different cancers. In CHECKMATE-9LA, a randomized open-label phase III trial in patients with metastatic or recurrent non-small cell lung cancer (NSCLC), nivolumab, ipilimumab, and chemotherapy (carboplatin, paclitaxel, pemetrexed, and cisplatin) combination was evaluated as a first-line treatment (NCT03215706). Median overall survival for combination group was 14.1 months compared to 10.7 months for chemotherapy alone. The overall response rate was 38% vs. 25% and median PFS was 6.8 months vs. 5 months [64]. Based on these results, FDA approved nivolumab plus ipilimumab and chemotherapy for first-line treatment of metastatic NSCLC on 26 May 26 2020 [64]. Pending results from multiple clinical trials evaluating the combination of anti-PD-1/PD-L1 plus anti-CTLA-4 with chemotherapies will inform whether such combinations are superior without a significant tradeoff with toxicities.

### 2.3. Limitations of PD-1/PD-L1 and CTLA-4 Checkpoint Inhibitors and Their Combinations

Despite research continuing to elucidate the efficacies of checkpoint inhibitors in treatments of cancers, the scope limitations are not entirely clear. Toxicities continue to be a major issue with the systematic use of checkpoint inhibitors [65]. Furthermore, while combination checkpoint blockade regimens have yielded better response rates and overall survivals, the tradeoff includes a significant increase in severe toxicities. A recent meta-analysis study was performed regarding the adverse drug reactions that can occur with anti PD-1 and PD-L1 along with anti-CTLA-4. From 2009 to early 2018, 613 fatal toxic events associated with immune checkpoint inhibitor (ICI) treatments were reported in the Vigilyze database (a database by the World Health Organization) [66]. There were 193 anti-CTLA-4-related deaths (mostly from colitis at 70%) and 333 deaths from anti-PD-1/PD-L1 treatments (mostly from pneumonitis at 33%). Deaths from a combination of anti-CTLA-4 and anti-PD-1 were mainly from colitis at 33% and 25% by myocarditis [66]. This is the largest ICI evaluation of fatal toxic events to date and these complications must be made aware. Through any treatment plan, the objectives are to improve the lifespan of patients while having minimal side effects during the process. Targeted delivery of checkpoint inhibitors may alleviate these systematic effects while maintaining or even improving the efficacies [65].

Other limitations of checkpoint inhibitor therapies include hyperprogression following checkpoint inhibition [67] and heterogeneous determinants of differences in response rates in patients with similar tumors [68]. Furthermore, utility and efficacy of checkpoint blockade is limited by several mechanisms of resistances through upregulation of additional checkpoint molecules, inherent lack of infiltration of immune effectors in the tumors, immunosuppressive and heterogeneous tumor microenvironments, and disparities in tumor-intrinsic factors such as oncogenic signaling pathways [69,70]. Some of these limitations are summarized in Table 1.

### 2.4. Emerging Checkpoint Molecules as Targets for Immunotherapy 

#### 2.4.1. T Cell Immunoglobulin and Mucin Domain 3 (Tim-3) 

Tim-3 is a protein of the Ig superfamily which is encoded by the gene hepatitis A virus cellular receptor 2 (*Havcr2*). Tim-3 is expressed on immune cells as well as non-immune cells. Ligands for Tim-3 include galectin-9 (Gal-9), high-mobility group protein B1 (HMGB1), phosphatidylserine (PtdSer), and carcinoembryonic antigen-related cell adhesion molecule 1 (CEACAM-1) [118]. The interaction of Tim-3 with its different ligands results in different effector consequences ranging from promotion of cross-presentation by dendritic cells (DCs), inhibition of innate immune responses to nucleic acids, induction of apoptosis of Th1 cells, and induction of T cell tolerance [119]. Based on its ability to induce immune exhaustion, Tim-3 is considered a negative regulatory checkpoint molecule [118,119]. Tim-3 has been shown to be highly expressed, and often co-expressed with PD-1 [120,121,122] on tumor infiltrating lymphocytes [118]. Additionally, Tim-3 has been shown to be highly expressed on T_regs_ and expression of Tim-3 by CD4^+^ T cells often correlates with poor outcome [119,123]. Tim-3 blockade has been shown to increase cytokine production by and proliferation of tumor-antigen specific T cells [120,121,124]. In preclinical models, inhibition of Tim-3 by anti-Tim-3 monoclonal antibodies, either as monotherapy or in combination with anti-PD-1 or anti-CTLA-4, has shown improved anti-tumor effects and growth inhibition [119,121].

In an in vitro study performed by Shayan et al., it was found that Tim-3 was upregulated in head and neck squamous cell carcinoma (HNSCC) tumor infiltrating lymphocytes (TILs) after PD-1 blockade, suggesting a potential mechanism of escape from or resistance to PD-1 blockade [122]. Interestingly, one study found that Tim-3 expression alone was not associated with exhausted phenotype of T cells and that coexpression of PD-1 and Tim-3 (or PD-1^hi^ Tim-3^-^) is associated with reduced cytokine production and proliferation [125]. These findings highlight the crosstalk between Tim-3 and PD-1 and emphasize the potential synergy of PD-1/Tim-3 blockade on T cell responses [125].

Multiple clinical trials evaluating the safety and efficacy of anti-Tim-3 antibodies, either as monotherapy or in combinations, are underway [126]. While some interim safety and efficacy results are available [127,128], completed results of safety profile and efficacies are not yet available. 

#### 2.4.2. V-Domain Ig Suppressor of T Cell Activation (VISTA)

VISTA is a transmembrane protein of the Ig superfamily that functions as an immune checkpoint involved with negative regulation of T cells [129,130]. VISTA is highly expressed on myeloid cells and it is expressed on both hematopoietic cells and cancer cells [131,132]. VISTA has been shown to be upregulated on myeloid-derived suppressor cells (MDSCs) from acute myeloid leukemia (AML) patients and that knockdown of VISTA on MDSCs decreased the inhibition of CD8^+^ T cell activity [132]. VISTA expressed on human tumor cells has been shown to suppress the proliferation and cytokine production by T cells in vitro and decrease the anti-tumor activity and tumor infiltration of CD8+ T cells in vivo in a mouse model (VISTA expressing ovarian cancer cell line) [131]. VISTA blockade in this model led to improved survival. 

Further pre-clinical studies have revealed that anti-VISTA therapy improves anti-tumor immunity and decreases tumor growth [133,134]. A study by LeMercier et al. evaluated the efficacy of VISTA blockade in murine models of melanoma and bladder cancers [134]. In both models, VISTA blockade inhibited tumor growth and improved proliferation, activation, and anti-tumor activity of T cells (increased IFNγ, granzyme B, CD107) [134]. Additionally, the authors showed that VISTA blockade decreases the induction of T_regs_ and reduces the suppressive capacity of natural T_regs_ [134]. Interestingly, VISTA mAb improved the proliferation of T cells in environments that contained or lacked T_regs_ [134]. These findings suggest the possibilities that VISTA may directly block the suppressive capacity of T_regs_ as well as confer naïve T cells resistance to Tregs’ mediated suppression [134]. Several VISTA antagonists are in clinical development; Curis announced FDA Clearance of IND Application for CI-8993 (First-In-Class Monoclonal Anti-VISTA Antibody) on 10 June with plans to initiate a Phase Ia/Ib study in the second half of 2020 [135].

### 2.5. Emerging Co-Stimulatory Molecules as Targets for Immunotherapy

#### 2.5.1. 4-1BB 

4-1BB, also called tumor necrosis factor receptor superfamily member 9 (TNFRSF9), is a transmembrane glycoprotein expressed on activated T cells, DCs, NK cells, monocytes, B cells, and neutrophils [87]. Ligation of 4-1BB with its ligand 4-1BB-L, expressed on activated APCs [136], aids in activation of all these cell types. The costimulatory 4-1BB molecule has been shown to influence the metabolic reprogramming of TILs whereby stimulation of 4-1BB improves metabolic sufficiency in endogenous and adoptively transferred CD8+ T cells [137]. In an experiment performed by Bukczynski et al., it was established that 4-1BB costimulation promotes expansion and activation of T cell memory responses to Epstein–Barr virus (EBV) and influenza virus [136]. In the study by Maus et al., artificial antigen presenting cells expressing ligands for 4-1BB (also expressing ligands for T cell receptor (TCR) and CD28) were shown to efficiently expand polyclonal and antigen specific CD8^+^ T cells ex vivo [138].

Several pre-clinical studies showed that agonists of 4-1BB, as monotherapies or in combination with other agents, result in potent anti-tumor activity against various tumors [84,85]. The clinical development, however, has been marred by toxicities and low efficacies. Two 4-1BB agonist antibodies (urelumab and utomilumab) have been tested in clinical trials. While urelumab has shown liver toxicities, utomilumab has shown limited toxicities but also lower efficacy [85]. Both of these agonists are still being tested (utomilumab predominantly), in combination with several other drugs, in different phase studies for different cancers (clinicaltrails.gov). While agonist antibodies to 4-1BB have not been a runaway success, the incorporation of 4-1BB in the CAR T cells has shown remarkable effects in the clinic [139,140,141]. Incorporation of 4-1BB (along with inducible T cell co-stimulator (ICOS)) in CAR T cells is thought to improve the persistence of these cells [142]. 

Since the combination of efficacy and safety profile of the two existing 4-1BB agonists (urelumab and utomilumab) has not been desirable, several efforts are being undertaken to improve efficacy while decreasing the toxicities [86,87]. These include, among others, the design of 4-1BB antibody with weak agonistic Fab coupled with an engineered Fc that has selective FcγRIIB binding [87] and design of trimeric 4-1BB ligand portion with capacity to engage 4-1BB on T cells but without the ability to crosslink FcγR (but retain FcRn binding) [86]. In the study by Claus et al., the trimeric 4-1BB ligand construct also included monovalent Fab fragment that binds to the tumor stromal component fibroblast activation protein (FAP) for efficient tumor targeting (FAP-4-1BBL) [86]. When combined with carcinoembryonic antigen-targeted T cell bispecific antibody (CEA-TCB), FAP-4-1BBL led to improved CD8+ T cell infiltration and tumor regression as compared to treatment with CEA-TCB alone in a murine model of gastric cancer (MKN45 cells xenografted with NIH/3T3-huFAP fibroblasts) [86]. It remains to be seen if the existing agonists or the new agonists under development, when used as monotherapies or in combination with other therapies, can result in significant response rates with manageable toxicities.

#### 2.5.2. OX40 

OX40, also called tumor necrosis factor receptor superfamily member 4 (TNFRSF4) and CD134, is a costimulatory molecule which belongs to the TNFR-superfamily of receptors. While constitutively expressed on T_regs_, OX40 is expressed on T cells upon antigen stimulation [143]. The ligand of OX40, OX40L, is found on B cells, epithelial cells, endothelial cells, activated APCs, NK cells, and activated T cells [88,144]. OX40/OX40L interaction results in activation and proliferation of T cells and also decreases the suppressive capacity of T_regs_ [145,146]; although one recent study has shown that OX40 agonist does not change intrinsic suppressive ability of T_regs_ [143]. As summarized by Fu et al., multiple preclinical studies of OX40 or OX40L agonists, either as monotherapies or in combination with other modalities, have shown improved anti-tumor responses, decreased tumor growth, and improved survival in different cancer models [88].

Multiple clinical trials, at various stages, are ongoing to evaluate the safety and efficacy of OX40/OX40L agonists, either as monotherapy or in combination with other treatments [88]. A preliminary analysis of dose escalation in phase 1 study of humanized IgG4 anti-OX40 antibody in patients with advanced solid tumors showed that this OX40 agonist was well tolerated with 16% of patients experiencing grade 3 TRAEs [89]. In another phase 1 dose escalation study of OX40 agonist in patients with advanced cancers, patients mostly experienced grade 1 and 2 toxicities and at least one metastatic lesion regressed in 12 out of 30 patients [90]. More safety and efficacy results from the ongoing trials will inform if OX40 agonism will add to the arsenal of therapies aimed at exploiting checkpoint molecules for cancer therapy.

### 2.6. Cellular Immunotherapy

**Figure 2:**
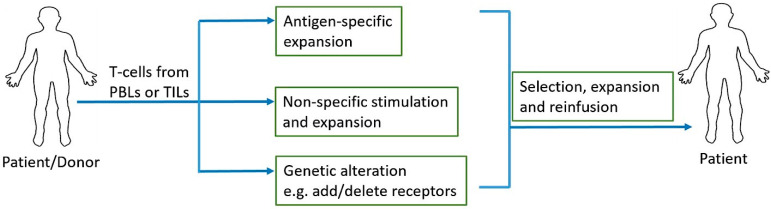
Adoptive T cell therapy. Peripheral blood lymphocytes (PBLs) or tumor infiltrating lymphocytes (TILs) are collected and antigen-specific or non-specific expansions can be performed depending on the source of T cells and their tumor antigen reactivity. Addition of antigen specific receptors and additions/deletions of stimulatory/inhibitory receptors can be achieved with genetic engineering prior to the expansion of T cells. Once desired quantity of these cells is reached through expansion, they are reinfused into the patient.

One of the most recent advancements in cancer immunotherapy has been the rise of cellular immunotherapy [147]. Cellular immunotherapy involves collection, modification, activation, expansion, and infusion of tumor-infiltrating lymphocytes, TCR engineered T cells, chimeric antigen receptor (CAR) T cells, or natural killer cells. Adoptive cell transfer (ACT) relies on extraction, expansion, and infusion of either TCR modified or chimeric antigen receptor (CAR) engineered T cells or endogenous tumor-specific T cells found at the malignant site [148] (Figure 2). Three major models of cellular therapy include tumor infiltrating lymphocytes (TILs), T cell receptors (TCRs) modified T cells, and CAR T cells [149]; in addition, NK cell therapy is also on the rise. In TIL therapy, tumor infiltrating T cells are extracted from tumors and selected and activated during ex vivo expansion before infusing them back into the patient. In TCR modified T cell and CAR T cell therapies, lymphocytes are acquired from the patient’s peripheral blood and they are genetically engineered to recognize and target tumor antigen(s) before infusing them back into the patient [148]. 

#### 2.6.1. Tumor Infiltrating Lymphocyte (TIL) Therapy and Combinations

TIL therapy was tested in metastatic melanoma patients as early as 1988, with results showing objective regression in 60% of patients who were previously untreated with IL-2 [150]. While some trials that followed achieved only modest response rates, subsequent trials with high intensity lymphodepletion resulted in improved response rates, objective response rate as high as 72% and 22% complete response [91], with TIL therapy in metastatic melanoma patients [91,92,93,94]. In a recent nonrandomized open-label phase II study of TIL therapy in metastatic melanoma patients, an overall response rate of 42% was achieved [95]. The overall response was 47% in checkpoint treatment-naïve patients and 38% and 33%, respectively for patients who had prior treatment with anti-CTLA-4 alone and anti-CTLA-4 plus anti-PD-1, respectively [95]. All patients in this study received nonmyeloablative lymphodepletion chemotherapy prior to TIL infusion.

TIL therapy has also been tested in combination with other drugs and as adjuvant therapy after surgery. In a long-term follow up (17-year median follow-up) of adjuvant TIL therapy in melanoma patients with regional lymph node metastases (but no detectable visceral metastases), the relapse rate for TIL treated patients was 75% compared to 84.09% for the control; and the median relapse-free survival was 14.2 months and 10.3 months, respectively [96]. With regards to combination, a pilot study evaluated the combination of vemurafenib (BRAF inhibitor) with ACT in 11 patients with metastatic melanoma harboring BRAF^V600E/K^ mutation [97]. An objective response rate of 64% and complete response of 18% was observed with the treatment [97]. Another study evaluated the safety and efficacy of ipilimumab combined with TIL therapy in 13 patients with metastatic melanoma [98]. At a follow up 12 weeks post-infusion, the objective response rate was 38.5% and median PFS was 7.3 months [98]. Currently multiple clinical trials are ongoing to evaluate the safety and efficacy of TIL therapy, as monotherapy or in combination with other treatments, in different cancers [151,152,153]. Results from these trials will inform if efficacy of TIL therapy, as monotherapy or as combination therapy, will extend to other cancers. 

#### 2.6.2. Chimeric Antigen Receptor (CAR) T Cell Therapy and Combinations 

**Figure 3:**
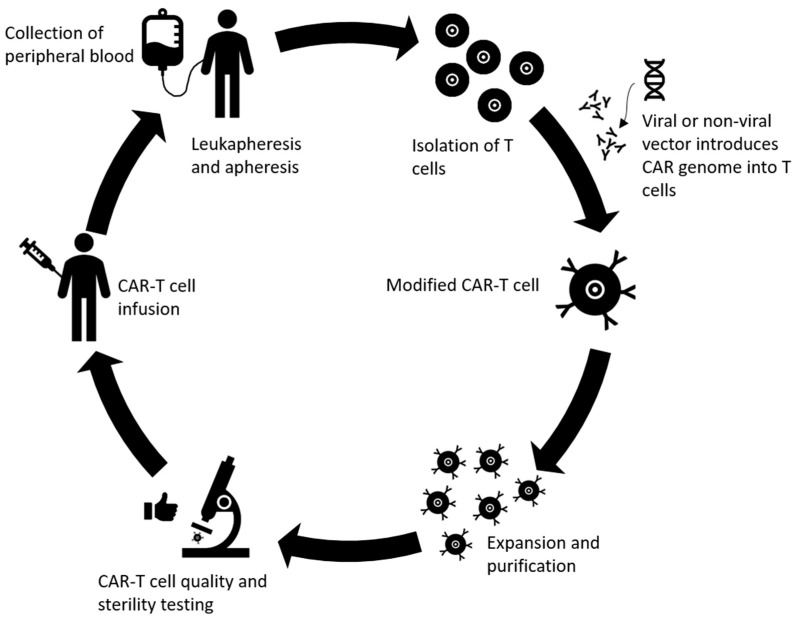
Overview of the production of chimeric antigen receptor (CAR) T cells. Peripheral blood is collected, T cells are extracted via leukapheresis and apheresis. The genome of the CAR is loaded onto the T cell using transduction from vectors (usually retroviruses). Next, the novel CAR T cells are expanded and purified until they reach sufficient numbers. The quality and overall immunological tolerance are tested for 2–4 weeks before reinfusing the cells into the patient.

CAR T cell therapy is another successful and promising development in the field of adoptive immunotherapy, where antigen receptor chimera are engineered into T lymphocytes for target recognition and effector functions independent of constraints of human leukocyte antigen (HLA) molecules [154]. CAR T cells, consisting of TCR constant domains fused to variable domains of anti-2,4,6-trinitrophenyl antibody, were first generated in 1989 [155]. Since then, both the constructs (different generations) and related effectiveness of CAR T cells have improved as different generations of CAR T cells are developed and tested in the clinic [156]. The process of producing CAR T cells is complicated and includes multiple steps [157]. First, white blood cells are separated from a sample of the patient’s peripheral blood along with stimulation with mAbs and/or cytokines to help isolate T cells from the sample [157,158]. After stimulation and expansion of T cells, genetic modification of the chimeric antigen receptor takes place. In the CAR, the TCR portion of T cells is replaced with antibody single-chain fragment specific toward a certain surface antigen of the tumor cell through non-viral or viral CAR vector and the cytoplasmic portion has a signaling component consisting of costimulatory domains such as CD28, 4-1BB, OX40, or CD40L and the CD3ζ signaling domain, depending on the different generations of the CAR T cells [154,158,159]. The fourth generation of CAR T cells even includes CAR inducible transgenic product such as cytokines [99]. Most of the time, a retroviral vector is used for the gene transfer [100]. The newly edited CAR T cells are then cultured to undergo expansion and proliferation to reach numbers adequate for infusion. Next, quality and sterility of the CAR T cells is tested and then the cells are infused into the patients [99]. A summary of these steps can be found in Figure 3. Advanced gene-editing technology known as clustered regularly interspaced short palindromic repeats (CRISPR)/Cas9, has also taken a front seat in the advancement of CAR T cell therapy. For example, generation of universal allogenic CAR T cells has been attempted by knocking out endogenous TCR and β2-microglobulin from the T cells [160]. Future studies can utilize this technology to knock in or knockout immunostimulatory or immunosuppressive genes of interest to improve response rates. Additionally, this can also be used to deliver or target gene products that reduce the risk of autoimmunity/toxicities.

CD19, a cell surface transmembrane protein expressed on B cells, is the most studied target for CAR T cells in the treatment of hematologic malignancies [99]. Therefore, CD19 targeted CAR T cells have been extensively evaluated for treatment of hematological cancers such as acute lymphoblastic leukemia (ALL), diffuse large B cell lymphoma, chronic lymphocytic leukemia, and B cell non-Hodgkin lymphomas [99,148]. Remission rates in hematologic malignancies after CD19 CAR T cell treatment have been reported to be as high as 93% [148]. In a study of CD19-specific CAR T cell treatment in children with relapsed or refractory ALL, 93% complete remission at one month after infusion and ~58% complete remission at a median follow up of 12 months after infusion were reported [102]. In another study (phase II, single-cohort, 25-center, global study; NCT02435849) of CD19 targeted CAR T cells in pediatric and young adult patients with refractory or relapsed B cell acute lymphoblastic leukemia (B-ALL), overall remission rate of 81% was reported at three months after infusion. In this study, the overall survival at 12 months was 76% [103]. In yet another phase I study, 16 patients with relapsed or refractory B-ALL were treated with CD19 CARs and a complete response rate of 88% was reported [104]. Unsurprisingly, such dramatic results in clinical trials have led to FDA approval of two CD19-targeted CAR T cell therapies (axicabtagene ciloleucel and tisagenlecleucel) [105].

Although CAR T cell therapy has been efficient in hematologic malignancies, relapses and toxicities such as cytokine release syndrome and neurotoxicities are often prevalent [106]. In patients with ALL that are treated with CD19 CAR, a relapse rate of 30–60% has been reported; where 10–20% of relapses are CD19 negative [107]. Unlike in hematological malignancies, the success of CAR T cell therapy has not extended to treatment of patients with solid tumors [148]. Non-exclusivity of tumor antigens has resulted in severe toxicities, including liver toxicities and multi-organ failure, associated with CAR T cell therapy in solid tumors [108,109,110,148]. CAR T cells that target relatively more specific tumor antigens in solid tumors have resulted in decreased toxicities, but without significant efficacies [148]. Additionally, antigen escape by the solid tumors provides another mechanism of resistance or low efficacy in solid tumors. In order to improve efficacy, a newer generation CAR T cell designs are aimed at prolonging the persistence of CAR T cells in vivo, augmenting effector responses by providing cytokine activation signals, exploiting inhibitory signals in the tumor microenvironment (TME) to generate stimulatory responses, and incorporating suicide genes for efficient shutdown of cells in cases of severe toxicities [148]. Multiple clinical trials are underway to evaluate the safety and efficacy of these newer generations CAR T cells.

The combination of CAR T cell therapy with checkpoint blockade has been studied [111,112,113]. In a small study of four children with relapsed B-ALL, the combination of pembrolizumab with CAR T cells resulted in prolonged persistence of CAR T cells and objective response was seen in two out of four children [111]. In another study of 14 patients with relapsed B-ALL or B lymphoblastic lymphoma, it was deemed that combination of pembrolizumab or nivolumab with CAR T cells is safe [112]. One case report of a patient with primary mediastinal large B cell lymphoma, that was refractory to CD19 targeted CAR T cells, indicated that the patient improved upon initiation of pembrolizumab on day 26 post-infusion of CAR T cells [114]. Multiple clinical trials are underway to evaluate the safety and efficacy of combination of CAR T cells with checkpoint blockade [113,153]. In a separate case report, a patient with Philadelphia chromosome-positive B-ALL, who relapsed after CD19 targeted CAR therapy, was treated with the combination of blinatumomab (bi-specific T cell engagers against CD19 and CD3) and ponatinib (multi-tyrosine kinase inhibitor) [115]. Remarkably, the patient responded and achieved complete remission lasting 12 months [115]. It remains to be seen, however, if concurrent combination of these drugs with CAR T cell therapy will result in similar efficacy. In another pilot study, 19 patients with CLL after ibrutinib (Bruton’s tyrosine kinase inhibitor) failure were treated with concurrent ibrutinib and CD19 CAR T cells [116]. The overall response rate in this study was 83% at four weeks. Compared to CAR T cell treatment without ibrutinib, concurrent treatment was associated with lower cytokine release syndrome severity without differences in in vivo CAR T cell expansion [116]. Trials with larger sample sizes and side-by-side comparison of sequential vs. concurrent treatment should inform the best approach for this combination to derive maximal benefit. Multiple studies evaluating the combination of CAR T cells with other therapeutic modalities are underway; hence results need to be awaited to determine if one or more of such combinations generate higher efficacies with manageable toxicities.

There are a variety of obstacles that ACT continues to face. There have been issues in defining what exactly constitutes a successful expansion of TILs. The extensive time for expansion to occur and immune competency of TIL cell lines from cultured cancer tissue raises concerns for efficacy [117]. In order for CAR T cells to be effective, successful migration, infiltration, and proliferation must endure long enough, in addition to overcoming immunosuppressive mechanisms, to employ an effective therapeutic response. There have been many factors hindering this, such as the lack of consistent targeting of intracellular antigens, tumor cell heterogeneity, loss of antigen specificity targets, and barriers to CAR T cell entry such as the blood–brain barrier [161]. Regardless of the obstacles, research and clinical trials continue to revolutionize the cellular immunotherapy landscape. 

## 3. Combination of Immunotherapy and Radiation Therapy

Radiation therapy is a key method for the local control of tumors that are unable to be surgically resected due to tumor location or the patient’s performance status or comorbidities. Radiation treatments deliver DNA-damaging X-rays or carbon-ion radiation into local tumor cells, causing direct cell damage and cell death [71,162]. Radiation also has the capacity to enhance T cell activation via upregulation of molecules within the tumor microenvironment such as major histocompatibility complex (MHC) class I molecules, tumor antigens, and interferon-γ [72]. When used in combination, radiation exposes tumor-specific antigens to trigger immune cell infiltration and activation, which can further be supplemented with immunotherapy agents. Together, this combination therapy has the power to elicit a synergistic immune effect in the control of tumor size and regression of metastases. Thus, the option of combined radiation with immunotherapy warrants studies to explore the efficacy, advantages, and possible treatment approaches for metastatic tumors. 

### 3.1. Mechanistic Effects of Radiotherapy Alone and in Combination with Immunotherapy

The tumor inhibitory mechanism of radiation is more than merely causing cellular DNA damage from high-energy beams. The immune system reacts to radiation-induced tumor antigen visibility and activation of pathways that trigger innate and adaptive immune responses [71]. While these are beneficial effects for the treatment of tumors, not all effects of radiotherapy are favorable for the elimination of cancerous cells. Negative effects can arise after radiation treatments. In addition to causing unwanted DNA damage to healthy cells near the exposure site, radiation can cause an upregulation in the cytokine transforming growth factor β (TGF-β) [163]. This cytokine causes a cascade of immunosuppressive actions, including a reduction of cytotoxic capability in CD8^+^ T cells, decreased differentiation of CD4^+^ T cells, and increased transcription of vascular endothelial growth factor (VEGF) [71]. Together this converts the tumor stromal microenvironment into a more immunosuppressive state [71]. While there are possible negative effects to radiation, the treatments offer benefits to local tumor control and long-term survival when used appropriately. 

**Figure 4:**
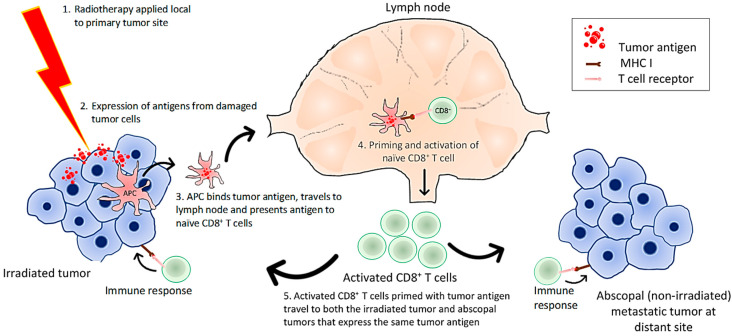
Mechanism of radiation-induced abscopal effect. Radiation damages tumor cells leading to generation of tumor antigens and neoantigens. Antigen presenting cells (APCs) can pick up these antigens, travel to draining lymph nodes, and present antigens and prime the naïve CD8^+^ T cells. The primed and activated T cells circulate to both the primary irradiated tumor and non-irradiated metastatic tumor sites and attack these tumors; hence generating the abscopal effect.

Combined radiation with immune-modulating agents has a greater potential to mediate tumor regression, especially in metastatic cancers [72]. A desired effect of this combination is to augment what is known as the abscopal effect, in which localized radiation of a tumor may induce tumor antigen recognition of distant metastatic tumors outside the field of radiation [73]. A simplified hypothesis is that the irradiated tumors release tumor antigens, which are recognized by APCs. APCs present tumor antigens from the irradiated tumor to CD8^+^ T cells in lymph nodes. The primed cytotoxic T cells undergo activation and proliferation. Tumor specific T cells can then circulate to distant non-irradiated tumors to initiate a cascade of recognition and elimination mechanisms [73,74] (Figure 4). Additionally, it has the ability to convert targeted approaches from local to systemic. 

The abscopal effect is rarely seen in patients who receive radiotherapy as a stand-alone treatment. This is likely because tumors possess altered antigen expression and mechanisms to confer immune tolerance, thereby evading host immune responses [73]. However, when an immunotherapy drug is added to the radiotherapy, studies have revealed that a much stronger and more sustainable T cell response is elicited [72]. Although the precise mechanisms have not been elucidated, it is thought that the radiation-induced immunogenic tumor cell death, along with increased release of chemokines, leads to activation and tumor-infiltration of new clones of T cells which can be targets for immunotherapy drugs such as ipilimumab to further enhance the antitumor immune responses [75,162]. This highlights the need and rationale of adding an immunotherapy agent into the radiation treatment plan to help prime and activate T cells to overcome immunological tolerance seen in tumors.

### 3.2. Efficacy of Radiotherapy Concomitant with Immunotherapy

While preclinical studies have shown that the combination of radiotherapy and immunotherapy results in improved responses, it is essential to determine the temporal relationship of radiotherapy and immunotherapy administration, the fractionation schedule of radiation, and the dosage of the immunotherapy agents that results in the best overall efficacy [76]. Concerns of toxicity levels should also be kept in mind when conducting these studies to reduce overlapping toxic effects of radiation and immunotherapy [77]. Among the various types of immunotherapy to combine with radiotherapy, ICIs are the most widely studied and have produced more robust clinical results [78]. Specifically, ICIs used to treat metastatic melanoma patients, anti-PD-1 and anti-CTLA-4, have shown encouraging results in combination with radiation therapy [76]. Other immune modulators such as vaccine immunotherapy, interleukins, and recombinant antigen technologies have not yet displayed substantial evidence of a clinical benefit when combined with radiotherapy [76]. 

Generally speaking, the toxicity of ICIs and radiotherapy appears to be well tolerated [79]. In a report summarizing the toxicities from studies evaluating the combination of radiotherapy with ICIs, it was reported that grade 3–5 toxicities range from 14–34% for combination with CTLA-4 inhibitors, and 5–10% for combination with PD-1 inhibitors. These rates are similar to the rates reported for ICIs as monotherapies [72]. Patients undergoing radiation treatment for cancers in the thoracic region should be monitored for radiation pneumonitis, an immune-mediated inflammatory reaction to damaged irradiated lung tissue [164]. Severe pneumonitis from radiation can be fatal; so the incidence of pneumonitis in combination therapy may be used as a benchmark to determine the radiation-dose limiting toxicity level [165]. Two notable limitations of using pneumonitis as the degree of toxicity in combination therapy are: (1) the occurrence of acute vs. delayed reaction pneumonitis and (2) the challenge of determining whether it is induced by the radiation or the immunotherapy agent [77]. The majority of phase I combination therapy trials use dose-escalation of radiation until the threshold of acute toxicity is reached. However, pneumonitis can still manifest several months post-radiation [77]. Therefore, a long follow-up period is recommended during prospective trials before toxicity can be determined and dose-escalation can continue. Multiple clinical trials are underway to identify the optimal timing of radiotherapy in conjunction with immunotherapies as well as dosing regimens [75,153] that results in the best efficacy. Results from these studies will help establish the relationship between the toxicity thresholds and the dosing and sequence of these two modalities.

## 4. Combination of Cancer Vaccines with Chemotherapy

Cancer vaccines seek to drive T cell activation by priming tumor-specific T cells with tumor-associated antigens [80]. A randomized phase II trial evaluated a cancer vaccine, consisting of multiple tumor-associated peptides, for renal cell carcinoma (RCC) after patients were treated with a single dose of cyclophosphamide to deplete T_regs_ [81]. Phase I of this study included 28 patients and phase II included 68 patients (HLA-A*02^+^). In the phase II study, a low-dose chemotherapy treatment of cyclophosphamide was administered three days before administration of the first dose of vaccine plus GM-CSF. Both phases assessed the responses of T cells in association with objective clinical benefits. While the PFS was not different between the groups (vaccine + GM-CSF + cyclophosphamide vs. vaccine + GM-CSF), the median overall survival was better in the group that was treated with cyclophosphamide (23.5 months vs. 14.8 months) [81]. While vaccine therapy has potential in combination therapy, challenges exist in the identification of optimal tumor antigen targets and therapeutic agents to counteract regulatory mechanisms that are antagonistic to immunotherapy.

Tumor protein p53 cancer vaccine has been studied in combination with chemotherapy [80]. p53 is a tumor suppressor gene that is often mutated in cancer cells and unlike its wild-type counterpart, it has a significantly longer half-life and is present in higher quantities in cancer cells, making it a suitable tumor antigen to exploit [80]. The goal of p53 cancer vaccination is to induce T cell responses against tumor-associated p53 peptides. In a study by Antonia et al., patients with extensive stage small cell lung cancer were treated with a combination of chemotherapy (cisplatin/VP-16, carboplatin/VP-16, or cisplatin/CPT-11) and p53 cancer vaccine (DCs with adenoviral transduction of p53) [80]. Patients were treated with first-line chemotherapy prior to receiving the p53 cancer vaccine, and 57.1% of patients developed p53-specific T cell responses. While most patients progressed despite the treatment with the vaccine, an objective clinical response of 61.9% was achieved when patients were treated with subsequent chemotherapy following the vaccination [80]. This suggests a synergistic relationship between the development of an antigen-specific response and clinical response to chemotherapy leading to improved patient prognosis; although treatment schedule (phased vs. concurrent) and doses will almost certainly have to be optimized to determine if there is significant benefit to such combinations. A combination of cancer vaccines with other chemotherapy agents such as gemcitabine, docetaxel, irinotecan, and platinum-based chemotherapies have been studied in clinic, with results demonstrating the added benefit of the combination with regards to response and survival rates [82].

Cancer vaccine combinations with chemotherapy face challenges in the clinical application of treatment. Factors such as immunosuppressive tumor microenvironment, advanced age, or disease stage can significantly affect patient outcomes [83]. Personalized combinations based on predictors of responses and tumor molecular phenotypes may aid in generating better responses and overcome some of the general challenges associated with the combination of cancer vaccines with chemotherapies [83].

## 5. Biomarkers in Immunotherapy and Combinations

There have been unprecedented response rates from combining therapies such as checkpoint inhibitors, vaccines, CAR T cells, and so forth. However, there are many patients who receive these treatments and do not respond. In addition, treatment-related adverse events are often prevalent and sometimes increase with the combinations. The identification of biomarkers will allow for personalized treatment with combinations resulting in improved responses while also avoiding severe toxicities. For example, between 2011 and April 2019, PD-L1 expression was deemed as a predictive biomarker in 28.9% of FDA approvals of immune checkpoint inhibitors and nine out of 45 approvals were linked to PD-L1 testing [166]. While exciting, this also highlights the challenges associated with the heterogeneity in the predictive power of the PD-L1 expression. Other challenges that lie ahead involve identifying precise immunotherapy mechanisms while simultaneously having less variability in the measurement, standardization, and analysis of assays using biomarkers [167].

A biomarker that is often used as of late is tumor mutational burden (TMB) [168]. TMB is a measure of the tumor genome’s somatic mutations found throughout the coding sequence. High TMB has been shown to associate with better response rates in patients [168]. For example, in a phase III trial of patients with NSCLC, it was found that patients with high TMB had a significantly higher PFS with ipilimumab and first-line nivolumab vs. with chemotherapy [54]. Patients with high TMB had a one-year PFS of 42.6% with ipilimumab and nivolumab vs. chemotherapy PFS of 13.2%. The median PFS was 7.2 months vs. 5.5 months for patients with high TMB and 3.2 months vs. 5.5 months for patients with low TMB for respective treatment with nivolumab plus ipilimumab vs. chemotherapy [54]. George et al. [169] and Havel et al. [170] summarized the current landscape of biomarkers associated with cancer immunotherapies.

## 6. Conclusions

Immunotherapies and their combinations with surgery, radiation, and chemotherapy have proven to be successful in some clinical studies. Their ongoing clinical developments and recent FDA approvals highlight their potential as powerful therapy tools to treat cancers as first-line or subsequent therapies. However, these therapies are still not efficacious in a wide range of patients and toxicity remains a major hurdle. Techniques to overcome drug-induced toxicity, subpar therapeutic responses, and lowered efficacy are currently being explored. Delivery technologies, targeted release mechanisms (with vehicles such as microparticles, liposomes, and nanoparticles), schedules of administration, and personalized combinations are currently under clinical evaluations to overcome drug-induced toxicities, subpar therapeutic response, and heterogeneity in responses in patients with similar tumors. Additionally, biomarker expression studies on tumors may help to define “hot tumors” (immune-responsive to therapy) and “cold tumors” (immune-resistant to therapy) in the coming years. Through innovative clinical designs and collaborations between interdisciplinary medical professions, new and enhanced treatments may be found and brought to the clinical realm to benefit patients.

## Figures and Tables

**Table 1:** Advantages and limitations of immunotherapies and their combinations.

Immunotherapy	Combination	Successes/Advantages	Limitations	References
Checkpoint Inhibitors	Checkpoint Inhibitors	Significantly improved response rates Long term disease eradication	High cost of treatment Dose limitation and increased grade treatment-related adverse events (TRAEs) Development of resistance Tumors lacking immune infiltrates may not respond Incomplete understanding of the determinants of hyperprogression	[11,29,52,53,54,55,56,57,58,59,65,66,67,68,69,70]
	Chemotherapy	Significantly improved response rates Increase in release of tumor-associated antigens and effector cells’ activation Some chemotherapies increase expression of checkpoint molecules, which can be overcome by combination with checkpoint inhibitors	Dose and sequence of combination is not universal Chemotherapy may inhibit activity of immune effectors Increased TRAEs	[30,31,32,33,35,36,50,64]
	Radiotherapy	Significantly improved response rates May help overcome resistance to checkpoint inhibition as monotherapy Radiotherapy leads to increased tumor antigen release, T cell activation/infiltration, and major histocompatibility complex (MHC) class I expression Increased abscopal effects	Dose-limiting toxicities prevalent Type of radiation, fractionation, and sequence of combination is not universal Variable results with combination in neoadjuvant, concurrent, and adjuvant settings Radiation may have direct negative effects on immune effectors and may increase frequency of T_regs_	[71,72,73,74,75,76,77,78,79]
Cancer Vaccines	Checkpoint Inhibitors	Improved response rates Vaccine induces anti-tumor immune effectors which can be acted upon by checkpoint inhibitors to improve immune responses	Most antigens that are the target of cancer vaccines are not tumor-restricted antigens; hence there is a risk of off-target effects Resistance through antigen escape and upregulation of additional checkpoint molecules is possible Not all studies have found added benefit of the combination, which highlights the need to design potent cancer vaccines No optimal dosing and sequence of treatments have been identified Unclear if the combination is efficacious in adjuvant or neo-adjuvant setting	[48,80,81,82,83]
Co-stimulatory Molecule Agonists	Checkpoint Inhibitors	Promote activation, and development and maintenance of T cell memory Multiple preclinical studies show improved activation of immune responses and anti-tumor effects of the combination	Existing trials of monotherapies show high toxicity or low efficacy Lack of available clinical trials results of combinations Unclear mechanisms for monotherapy or combinations Timing of treatment for optimal efficacy is unclear	[84,85,86,87,88,89,90]
Tumor Infiltrating Lymphocyte (TIL) and Chimeric Antigen Receptor (CAR) T Cell Therapy	Checkpoint Inhibitors, Chemotherapy	Unprecedented response rates, including high frequency of complete responses High response rates even in patients who have failed multiple prior therapies Two CD19 CAR T cells are already U.S. Food and Drug Administration (FDA)-approved Amenable to modulation of T cell function to improve efficacy, decrease off-target effects, and decrease toxicity CAR T cell therapy is not MHC-restricted Combination helps overcome exhaustion	Significant risk of off target effects and toxicities Neurotoxicity and cytokine release syndromes are challenges that still needs to be overcome Lengthy and stringent manufacturing process Lack of sufficient trials evaluating the feasibility, dosing sequence, and toxicities associated with the combinations Treatment is expensive In vivo persistence of infused cells are not optimal	[91,92,93,94,95,96,97,98,99,100,101,102,103,104,105,106,107,108,109,110,111,112,113,114,115,116,117]

## Abbreviations

ACT	Adoptive cellular therapy
APC	Antigen presenting cell
CAR	Chimeric antigen receptor
CDK	Cyclin-dependent kinase
CI	Confidence interval
CML	Chronic myeloid leukemia
CRISPR	Clustered regularly interspaced short palindromic repeats
CTL	Cytotoxic T-lymphocyte
CTLA-4	Cytotoxic T-lymphocyte-associated protein 4
DC	Dendritic cell
DNA	Deoxyribonucleic acid
FasL	Fas ligand
HLA	Human leukocyte antigen
ICI	Immune checkpoint inhibitor
IFN	Interferon
Ig	Immunoglobulin
IL	Interleukin
ITIM	Immunoreceptor tyrosine-based inhibition motif
LAG-3	Lymphocyte activation gene 3 protein
LFT	Liver function test
mAbs	Monoclonal antibodies
MHC	Major histocompatibility complex
NF-kB	Nuclear factor kB
NHL	Non-Hodgkin’s lymphoma
NK	Natural killer
NSCLC	Non-small cell lung cancer
OS	Overall survival
pCR	Pathologic complete response rates
PD-1	Programmed cell death protein 1
PD-L1	Programmed death-ligand 1
PFS	Progression free survival
RCC	Renal cell carcinoma
TCR	T cell receptor
TGF	Transforming growth factor
TIL	Tumor infiltrating lymphocyte
TIM-3	T cell Ig mucin domain-containing 3
TME	Tumor microenvironment
TNBC	Triple negative breast cancer
TNF	Tumor necrosis factor
TNFR	Tumor necrosis factor receptor
TNFRSF4	Tumor necrosis factor receptor superfamily member 4
TRAEs	Treatment-related adverse effects
Treg	Regulatory T cell
VEGF	Vascular endothelial growth factor
VEGFR	Vascular endothelial growth factor receptor
VISTA	V-domain Ig suppressor of t-cell activation
